# Discovery of Putative Small Non-Coding RNAs from the Obligate Intracellular Bacterium *Wolbachia pipientis*

**DOI:** 10.1371/journal.pone.0118595

**Published:** 2015-03-04

**Authors:** Megan Woolfit, Manjula Algama, Jonathan M. Keith, Elizabeth A. McGraw, Jean Popovici

**Affiliations:** 1 School of Biological Sciences, Monash University, Clayton, Victoria, Australia; 2 School of Mathematical Sciences, Monash University, Victoria, Australia; International Atomic Energy Agency, AUSTRIA

## Abstract

*Wolbachia pipientis* is an endosymbiotic bacterium that induces a wide range of effects in its insect hosts, including manipulation of reproduction and protection against pathogens. Little is known of the molecular mechanisms underlying the insect-*Wolbachia* interaction, though it is likely to be mediated via the secretion of proteins or other factors. There is an increasing amount of evidence that bacteria regulate many cellular processes, including secretion of virulence factors, using small non-coding RNAs (sRNAs), but sRNAs have not previously been described from *Wolbachia*. We have used two independent approaches, one based on comparative genomics and the other using RNA-Seq data generated for gene expression studies, to identify candidate sRNAs in *Wolbachia*. We experimentally characterized the expression of one of these candidates in four *Wolbachia* strains, and showed that it is differentially regulated in different host tissues and sexes. Given the roles played by sRNAs in other host-associated bacteria, the conservation of the candidate sRNAs between different *Wolbachia* strains, and the sex- and tissue-specific differential regulation we have identified, we hypothesise that sRNAs may play a significant role in the biology of *Wolbachia*, and in particular in its interactions with its host.

## Introduction

*Wolbachia pipientis* is a vertically transmitted endosymbiotic Alphaproteobacteria that is thought to infect up to 40% of arthropod species [1]. Different *Wolbachia* strains induce a diverse range of effects in their hosts, including multiple forms of reproductive manipulation that enhance transmission of the endosymbiont to the next host generation [2,3]. More recently it has also been discovered that a number of *Wolbachia* strains inhibit the replication of viral and other pathogens in both their natural hosts, such as *Drosophila melanogaster*, and heterologous hosts such as *Aedes aegypti* [4–6]. These effects make *Wolbachia* an attractive biocontrol agent for vector-borne diseases, and field releases of *Wolbachia*-infected *A*. *aegypti* are currently being tested in trials with the aim of reducing dengue virus transmission [7].

The molecular mechanisms by which *Wolbachia* causes these different host phenotypes remain largely unknown. Recent work has demonstrated that *Wolbachia* infection modulates expression of mosquito host miRNAs that regulate diverse genetic targets, including host metalloprotease and methylase genes [8–10]. *Wolbachia* infection in other taxa has also been shown to affect transcription of host genes involved in iron metabolism and the oxidative stress response [11–13]. At least some host responses to *Wolbachia* infection are likely to be induced by effectors secreted by the endosymbiont. *Wolbachia* has a conserved and functional type IV secretion system (T4SS) [14], and these systems are known to play a role in infection, survival and proliferation in many other symbiotic and pathogenic intracellular prokaryotes [15]. *Wolbachia* genomes also contain an unusual number of genes encoding ankyrin domains. Host-interacting ankyrin proteins are secreted via the T4SS in other intracellular Alphaproteobacteria such as *Anaplasma phagocytophilum* and *Ehrlichia chaffeensis*, and these proteins are considered the most likely candidates to underlie the molecular dialogue between *Wolbachia* and its host [16–20].

Numerous *Wolbachia* genes, including those encoding ankyrin domains, show host sex- and tissue-specific expression patterns [21,22], further suggesting that they may be involved in host interaction. The mechanisms by which *Wolbachia* regulates the expression of these genes are currently unknown. Few transcription factors have been identified in *Wolbachia* genomes, and these factors have so far been shown to regulate only a small number of genes [23]. Recently, however, numerous other species of facultative or obligate intracellular bacteria have been shown to use small non-coding RNAs (sRNAs) to regulate the expression of genes associated with diverse aspects of host interaction, including iron homeostasis [24], the cell cycle [25], quorum-sensing [26], secretion systems [27] and secreted virulence factors [28–30]. These small RNAs are highly variable in sequence and function, and vary in number from a few tens to a few hundreds in many bacterial genomes [31].

There are at least five main classes of sRNAs, which regulate gene expression in several ways [31,32]. Antisense sRNAs are typically 50–500 nt in length, are transcribed from the opposite strand of the genes that they regulate, and act via extensive complementarity with their target mRNAs. Trans-encoded sRNAs, in contrast, are often shorter (around 100 nt), are usually encoded intergenically or with partial overlap of one or more CDSs, may regulate many different mRNAs, and have much more limited complementarity with their targets. Both antisense and trans-encoded sRNAs may interact with mRNA targets to enhance or inhibit translation. A third kind of sRNA, also encoded outside CDSs, are 5' riboswitches, which do not operate as independent transcripts but are part of the mRNA they regulate. Fourth, there are a small number of sRNAs, such as 6S sRNA, that interact with proteins rather than mRNA. Finally, bacteria also encode a number of 'housekeeping' sRNAs that do not pair with mRNAs or regulate proteins; these include the ribozyme RNase P, the 4.5S RNA component of the signal recognition peptide, and tmRNA. Genes encoding tmRNA, 4.5S sRNA, RNase P and 6S sRNA are present in *Wolbachia* genomes, and the latter two show host tissue-specific expression in filarial nematodes [21]. To our knowledge, however, no antisense or trans-encoded sRNAs have previously been identified in *Wolbachia* genomes.

The majority of trans-encoded sRNAs described to date are expressed under specific growth conditions [31], and this class of sRNA may therefore be of particular interest in elucidating host sex- and tissue-specific gene regulation in *Wolbachia*. In this study, two independent methods have allowed the identification of candidate trans-encoded sRNA in several *Wolbachia* strains. The first method is based on examination of RNA-Seq data from the *Wolbachia* strains *w*MelPop, *w*MelPop-CLA and *w*MelCS. To investigate the potential utility of RNA-Seq for *Wolbachia* gene expression studies, we had previously performed a number of trial runs of this sequencing technology. These data were not ideal for detection of sRNAs, as we did not perform strand-specific sequencing and had chosen to sequence DNA fragments of ∼300 nt, which is longer than many known sRNAs. Despite these limitations, however, we serendipitously identified a number of sRNA candidates while analysing the sequencing reads for other purposes. The second method we used to identify candidate sRNAs is bioinformatic, and based on comparative genomics of the strains *w*Mel and *w*Pip.

To increase the probability that the candidates identified using the methods above are true sRNAs, we have conservatively focused on transcripts that are encoded entirely within intergenic regions rather than overlapping a CDS, and that are transcribed specifically rather than as an intergenic component of a polycistronic mRNA. We identified several candidate sRNAs, and have experimentally confirmed the differential expression of one putative sRNA in four strains of *Wolbachia*, and in different host sexes and tissues.

## Materials and Methods

### Fly rearing and cell culture

*Drosophila melanogaster* (*w* ^*1118*^) stock lines stably infected with the *w*Mel, *w*MelPop, *w*MelCS and *w*Au strains of *Wolbachia* were maintained on standard molasses and cornmeal medium at a constant temperature of 25°C with a 12h light/dark cycle [33,34].

C6/36 cells infected with *w*MelPop-CLA were routinely passaged in RPMI 1640 medium supplemented with 10% FBS [35].

### Sample preparation for RNA-Seq experiment

We performed RNA-Seq sequencing on five trial libraries. Three libraries were created using material from C6/36 cells infected with *w*MelPop-CLA, and two libraries were created from the heads of flies infected with either *w*MelPop or *w*MelCS. In an attempt to minimize the number of experimental manipulations that could affect the transcriptomic profile, we created the two fly libraries without performing either purification of *Wolbachia* from the host material or depletion of host or *Wolbachia* rRNA. For each of these libraries, we dissected the heads from 10 flies. *w*MelCS was obtained from *D*. *melanogaster* Canton S virgin female flies at 3 days of age, and *w*MelPop was obtained from *D*. *melanogaster w* ^1118^ virgin female flies at 3 days of age. In each case, total RNA was isolated after homogenization of dissected heads in 100ul of Trizol (Invitrogen). RNA was then purified according to the manufacturer’s instructions and DNase-treated (DNase I recombinant, Roche) before being sent for Illumina sequencing.

The three samples derived from *w*MelPop-CLA-infected cell culture were each subject to different treatment. For the first, total RNA was isolated from a 175cm^2^ flask of *w*MelPop-CLA-infected C6/36 cells at ∼80% confluence using Trizol according to the manufacturer’s instructions, and the RNA was DNase treated and sent for Illumina sequencing. For the second sample, we extracted total RNA from a single flask of cell culture as above, while for the third sample, we purified *Wolbachia* from the cell culture using the method of Iturbe-Ormaetxe et al [36], then performed total RNA extraction. For both samples, RNA was DNase-treated and then depleted for host and bacterial rRNA using successively the RiboMinus Eukaryote kit (Ambion) and the MicrobExpress bacterial mRNA Enrichment kit (Ambion) according to the manufacturer’s instructions. After depletion, first and second-strand cDNA synthesis was done using SuperScript III Reverse Transcriptase (Life Technologies) and DNA Polymerase I, Klenow Fragment (NEB) according to the manufacturer’s instructions. cDNAs were purified using the MinElute Reaction Cleanup Kit (Qiagen) before being sent for Illumina sequencing.

The second and third cell culture samples (those with rRNA depletion) were indexed on a single lane of Illumina GAII, and sequenced at Micromon (Monash University), with 300 bp size selection and 75 bp paired-end reads. The remaining three samples were sent to Macrogen (South Korea) for library preparation and sequencing indexed on a single lane of HiSeq, with 300 bp size selection and 70 bp paired-end reads.

The RNA-Seq sequence data have been deposited at NCBI under Bioproject PRJNA266744, sample numbers SAMN03174110, SAMN03174111, SAMN03174113, SAMN03174115 and SAMN03174116.

### Data analysis and mapping of RNA-Seq reads

We filtered reads for quality using Trimmomatic [37] by removing all trailing bases with quality less than 30, and then discarding (1) reads shorter than 40 nt after trimming and (2) any unpaired reads. We then performed read mapping and downstream analyses using the Nesoni toolset (http://www.vicbioinformatics.com/software.nesoni.shtml). Filtered paired reads were mapped to the reference *w*Mel genome [18] using BWA [38], and then mappings were filtered so that read pairs with multiple equally good alignments were randomly assigned to one of those alignments. We then created a modified *w*Mel gff file that listed intergenic regions as well as the more typical annotation features (CDSs, rRNAs, tRNAs, etc), and used a custom Perl script to count the alignments to each feature.

Because some intergenic regions are smaller than the mean fragment size sequenced, and because there appears to be a substantial amount of polycistronic transcription occuring in *w*Mel, many "intergenic" mapping counts actually reflect transcription of flanking genes. Nonetheless, these counts provided us with a preliminary list of candidate intergenic regions with high transcription levels. We then inspected the read mapping data for these candidate regions in the Artemis genome browser [39]. We identified regions that appeared to have intergenic-specific transcription, based on mapping of read pairs, for further investigation.

### Change-point analysis: prediction of conserved candidate sRNAs

We used the program *changept* to identify a class of segments characterized by a high degree of conservation. The process followed in this analysis is described below.

#### Sequence and alignment of data

We used the published complete genome sequences of *w*Mel and *w*Pip (NCBI accession numbers NC_002978.6 and NC_010981.1 respectively) to identify intergenic regions that were highly conserved between these strains. Fragments of the genome may show low levels of divergence between strains for at least two reasons. The first possibility is that they are evolving under selective constraint, and these are the regions we wish to identify. Alternatively, however, genomic regions that were horizontally transferred between *w*Mel and *w*Pip after the divergence of these strains will also be more similar to one another than expected, not due to selection but because they have a more recent common ancestor than the rest of the genome. We took two approaches to exclude these regions. First, we masked prophage regions [40] and a known region of horizontal gene transfer (WD0507-WD0517 [41]) in the genomes before analysis. Secondly, we also performed a post hoc check for horizontal transfer after candidates were identified by extracting their nucleotide sequences from the *w*Mel genome and using them as megablast queries against the NCBI NT database. To attempt to exclude regions that have artifactually low levels of divergence due to recent horizontal transfer between supergroups, we accepted only those candidate regions that had better hits to all available A group genomes than to all available B group genomes. The *changept* procedure we used to identify these conserved non-CDSs is described below. We aligned the masked genomes using progressive Mauve [42], and used the accessory script stripSubsetLCBs (available from http://gel.ahabs.wisc.edu/mauve/snapshots/) to extract local colinearity blocks (aligned core genome blocks) at least 500 nt in length. We then used a custom script to convert this XMFA output file into Fasta format.

#### Data transformation

The pairwise alignment of *w*Mel and *w*Pip was then converted into a *changept* input sequence using a 16-character code (A = (a, b, c, d, e, f, g, h, i, j, k, l, m, n, o, p)), (Table 1). Insertions and deletions were excluded. The alignment blocks were separated using the ‘#’ symbol; these are considered as fixed change-points by the model. In 16-character representation, characters ‘a’, ‘f’, ‘k’ and ‘p’ represent conserved bases. Although this sequence also contains other biologically significant information, such as the GC content of each species and transition/transversion ratio, we were mainly focused on the different levels of conservation between *w*Mel and *w*Pip.

**Table 1:** *changept* 16-character code used for conversion of pairwise alignment of *w*Mel and *w*Pip.

*w*Mel	A	A	A	A	C	C	C	C	G	G	G	G	T	T	T	T
*w*Pip	A	C	G	T	A	C	G	T	A	C	G	T	A	C	G	T
Symbol	a	b	C	d	e	f	g	h	i	j	k	l	m	n	o	p

#### Model selection

*Changept* currently requires the user to specify the number of segment classes. Separate segmentation analyses were performed for models with 1–12 segment classes. Each model was run for 1,000 iterations. Selecting the model with the most appropriate number of classes involved model selection criteria discussed in [43]. In summary, we calculated approximations to three information criteria: AIC, BIC and DIC (Figure A in S1 File). The model with the smallest information criterion is generally considered optimal. However a subjective judgment was made on which model to choose; models containing classes with very low mixture proportions were considered to be over-fitted and thus a model with fewer classes was selected. For this data, we selected the 7-class model. BIC increases with the number of classes, displaying an unusual behaviour (Panel B, Figure A in S1 File); therefore we based our decision on AIC and DICV (Panel A, Figure A in S1 File). The first local minimum of both these criteria occurred at seven classes.

#### The most conserved class

To determine the most conserved class of the selected model, the mean proportion of alignment matches was calculated for each iteration of the sampler:

$$E(\theta )\;=\;\frac{\theta_{a}+\theta_{f}+\theta_{k}+\theta_{p}}{\sum_{j\in A}\theta_{j}}$$

where *θ_j_; j ∈ A* is the frequency of character *j* in the most conserved segment class.

These values were plotted against iteration number (Figure B in S1 File). This plot was also used as an added check to determine if the model had converged along with the loglikelihood plot (obtained by plotting the log likelihood at each iteration for the 1000 iterations).

#### Calculating profile values and generating wiggle track

**Fig 1:**
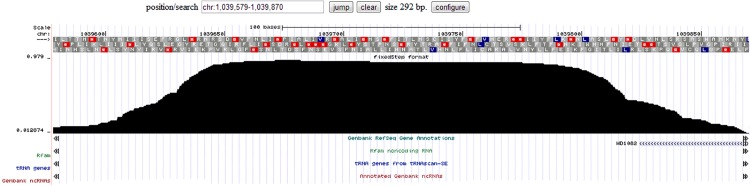
The WIG profile of a highly conserved feature. This profile shows the probability that each position in the region belongs to the most conserved class. The conserved non-coding region is positioned in wMel coordinates 1,039,579–1,039,870. The top 3 bars containing single letter amino acid codes show 3 possible protein translation phases. At the bottom, coding region WD1082 is shown (in light blue with arrows). There are no previously annotated non-coding regions corresponding to this region.

We used the program *readcp* (part of the *changept* package) to calculate profile values for the most conserved segment class. The profile shows the probability that each sequence position belongs to the specified class. These posterior probabilities are estimated using the samples from the post burn-in phase (that is, the first 150 samples were discarded) by Monte Carlo integration. A complete description of the *changept* and *readcp* programs can be found in [44,45]. The *readcp* output is then used to generate a wiggle track (https://cgwb.nci.nih.gov/goldenPath/help/wiggle.html). This file was uploaded to the UCSC browser (http://microbes.ucsc.edu/) for viewing in order to identify highly conserved non-coding segments in *w*Mel and *w*Pip. A section of the wiggle track corresponding to one of the candidate sRNAs (*w*Mel coordinates 1,039,579–1,039,870) predicted by the *changept* program is shown in Fig. 1. The non-CDs regions longer than 50 nt with profile value ≥ 0.5 are considered as most likely candidates.

### Validation of 5′ ends of candidate sRNAs by Rapid Amplification of cDNA Ends

A Rapid Amplification of cDNA Ends (RACE) procedure was used to determine the 5’ ends of a selection of candidate sRNAs [46]. The protocol used is based on the procedure described by [47]. Briefly, total RNA was isolated from whole bodies of ∼100 *w*Mel-infected 1-day-old adult male *w* ^*1118*^ flies using Trizol, DNAse-treated, and treated with Tobacco Acid Pyrophosphatase (Epicentre) for 30 min at 37°C. T4 RNA ligase (NEB) was used to add a short RNA adaptor on the 5’ ends of RNA. Reverse transcription of RNA was performed using primers complementary for candidate sRNAs and Superscript III (Invitrogen). Primers and adaptor sequences are listed in Table A in S1 File. The resulting cDNA was used as template for PCR using candidate sRNA specific primers and RNA adaptor specific primers. PCR products were run on a 1% agarose gel, gel-extracted using the QIAEX II kit (QIAGEN), and cloned in pGEMTeasy vector (Promega). *E*. *coli* DH5α were transformed with the constructs and plasmids were purified using the QIAprep spin miniprep kit (QIAGEN) prior to sequencing using SP6 primer (Micromon, Monash University, Australia).

### Verification of transcription of candidate sRNA within intergenic regions

In order to verify that expression in each intergenic region was due to the transcription of the candidate sRNA itself and not the result of transcription together with a downstream gene in a single RNA molecule, a set of RT-PCRs were done using primers overlapping genes downstream of the intergenic regions. These “overlapping RT-PCRs” were done to verify that the 3’ end of a 5’RACE-confirmed candidate sRNA was indeed within the intergenic region. PCR was performed on cDNA from *w*Mel-infected *w* ^*1118*^ 1 day-old female abdomens and ovaries. Briefly, total RNA was isolated from pools of 5 abdomens or pools of 10 dissected ovaries using Trizol. RNA was further purified according to Trizol instructions and DNase treated. cDNAs were synthesized from 1 μg of total RNA using random primers and SuperScript III, in accordance with the manufacturer's instructions. PCR was performed on cDNA and for every primer pair on DNA controls. PCR products were then visualised on a 2% agarose gel. All primers used are listed in Table A in S1 File.

### RNA extraction, cDNA synthesis and quantitative PCR analysis of candidate sRNA

All RNA extractions were performed on 1-day-old flies. Three types of biological material were used for RNA extraction and further qRT-PCR analysis: whole bodies of male flies, abdomens of male and female flies, and dissected tissues (head, carcass, gonads) of male and female flies. Total RNA was isolated from individual whole bodies of male *w* ^*1118*^ flies infected with *w*Mel, *w*MelCS, *w*MelPop or *w*Au strains (n = 15 individuals per line) using Trizol. Total RNA was isolated from pools of 5 abdomens of either male or female *w* ^*1118*^ flies infected with *w*Mel, *w*MelCS, *w*MelPop or *w*Au strains (n = 15 pools per line) using Trizol. Total RNA was isolated from pools of dissected tissues of 10 male or female *w* ^*1118*^ flies infected with *w*Mel (n = 12 pools per tissue per sex). Flies were ice-anaesthetized, then head, gonad and carcass were dissected in ice-cold PBS and immediately transferred into Trizol. All RNA samples were further purified according to Trizol instructions and DNase treated. cDNAs were synthesized from 1 μg of total RNA using random primers and SuperScript III, in accordance with the manufacturer's instructions. Expression of candidate sRNA in all samples was measured by qPCR using the LightCycler480 SYBR Green I Master (Roche) on a LightCycler480 II instrument (Roche) in duplicate on a 2–5 times dilution of the cDNAs. Primers are listed in Table A in S1 File. *Wolbachia* surface protein *wsp* expression was used as reference to normalize candidate sRNA expression and account for *Wolbachia* density [48]. On a subset of samples, to confirm differential expression, we compared the use of *wsp* as reference gene to *Wolbachia* 16S and *Drosophila melanogaster rps17*. Relative quantification of expression was calculated using the LightCycler480 II software. Significant differences in candidate sRNA expression were tested by Mann-Whitney *U* test using GraphPad Prism 5 software (GraphPad Software, San Diego, California USA).

## Results

We used two approaches to identify candidate novel sRNAs in *Wolbachia*. For the first approach, we extracted RNA from *D*. *melanogaster* flies or *Aedes albopictus* C6/36 cell lines infected with *Wolbachia*, performed RNA-Seq, and mapped the resulting reads to the *w*Mel and host genomes (Table 2). As these sequencing runs were exploratory trials and our treatments (rRNA depletion and *Wolbachia* purification) were not replicated, we cannot draw any firm conclusions about the effects of each treatment on the quality or content of the resulting sequence data. It is clear, however, that some kind of purification, rRNA and/or host RNA depletion is necessary to obtain reasonable coverage of the *Wolbachia* transcriptome without wasted sequencing effort as shown in other studies [49,50]. Even though our RNA-Seq experiments were not designed for this purpose, we observed reads mapping to intergenic regions of *Wolbachia* (S1 Data). Given the variable coverage of the *Wolbachia* genome we obtained from the different RNA-Seq experiments, we have focused our work using the dataset with the highest number of reads mapping to *Wolbachia*: RNA extracted from *Wolbachia* in cell culture.

**Table 2:** Summary of the RNA-Seq data obtained.

Source material	Cells	Cells	Cells	Fly head	Fly head
Treatment	Purification and rRNA depletion	rRNA depletion	Untreated	Untreated	Untreated
*Wolbachia* strain	wMelPop-CLA	wMelPop-CLA	wMelPop-CLA	wMelPop	wMelCS
Sequencing instrument	GAII	GAII	HiSeq	HiSeq	HiSeq
Number of filtered reads	31,348,758	18,079,262	135,928,880	130,923,592	132,780,000
Number of mapped reads	27,244,266	12,094,331	119,609,644	129,883,646	131,671,602
Number mapping to *Wolbachia*	22,734,365	932,604	21,641,258	4,176,245	1,061,022
(% of mapped reads)	(83%)	(8%)	(18%)	(3%)	(1%)
Number mapping to host	4509901	11161727	97968386	125707401	130610580
(% of mapped reads)	(17%)	(92%)	(82%)	(97%)	(99%)
Number mapping to host rRNA	4276896	10822638	97535071	122477846	126476269
(% of mapped reads)	(16%)	(89%)	(82%)	(94%)	(96%)
(% of host reads)	(95%)	(97%)	(99.5%)	(97%)	(97%)
Among reads mapped to *Wolbachia*:					
16S	13%	9%	44%	44%	43%
23S-5S	79%	54%	50%	51%	53%
CDS	8%	37%	6%	5%	4%
% of CDS with > 10 reads	95%	83%	94%	74%	49%

### RNA-Seq reveals transcription from multiple *Wolbachia* intergenic regions

A large number of RNA-Seq reads from each sequencing library mapped to intergenic regions of the *w*Mel reference genome, as listed in S1 Data. However, many of these reads may derive not from independent intergenic transcripts, but rather from 5' or 3' UTRs, or the intergenic regions of polycistronic transcripts. We identified candidate sRNAs by selecting intergenic regions with high transcription levels, and then inspecting the mapping of paired reads in these regions. We excluded from further analysis transcribed intergenic regions in which one read of any pair mapped to the intergenic region and the other mapped to a flanking CDS, and focused only on those regions in which both ends of all read pairs mapped within the intergenic region. Candidate sRNAs presented here were identified using the data obtained from *w*MelPop-CLA in C6/36 cells.

### Bioinformatic prediction of candidate conserved intergenic sRNAs

Our second, independent approach to identifying candidate sRNAs was based on comparative genomics. Previous work has demonstrated that, while some sRNAs are specific to a single bacterial strain or species, others show conservation of sequence across broader taxonomic distances [51,52]. If conserved intergenic sRNAs are undergoing purifying selection to maintain functionality, we would expect them to evolve more slowly than other intergenic regions that are not functionally constrained in this way. To search for such regions, we aligned the published genome sequences of the moderately divergent *Wolbachia* strains *w*Mel and *w*Pip (from the A and B supergroups of *Wolbachia*, respectively [18,53]), and used the program *changept* to identify highly conserved non-CDS regions. A full description of the *changept* model can be found in previous papers [54–56]. In summary, the algorithm takes as input a sequence of characters (which may represent pairwise or multiple alignments) and estimates positions (called change-points) that delineate homogeneous segments. Using the *changept* program, we identified a class of segments (Class 4 of the 7-Class model, Figure B in S1 File) characterized by the highest degree of conservation (∼95% between *w*Mel and *w*Pip). Non-coding segments longer than 50 nt and with ≥ 0.5 probability of belonging to this class form the focus of our analysis. There are 42 non-CDS regions in the most conserved class (Table B in S1 File). These included the 16S, 23S and 5S rRNA genes, 17 tRNA genes, a recent pseudogene (WD0002), and the housekeeping sRNAs RNase P and tmRNA. We also identified 19 highly conserved intergenic regions (Table 3) with no previous annotation, which represented a preliminary list of candidate conserved sRNAs.

**Table 3:** Intergenic regions predicted by *changept* to be highly conserved.

Coordinates in *w*Mel genome	Upstream/downstream CDS	Length (nt)
44,380–44,468	dnaJ/tRNA-Arg-1	89
85,867–85,929	dprA/WD0093	63
279,526–279,619	WD0299/coxB	94
547,479–547,732	nuoD/WD0562	254
611,202–611,370	WD0625/WD0626	169
612,281–612,391	WD0626/WD0627	111
622,779–622,923	WD0632/WD0633	145
623,094–623,293	WD0632/WD0633	200
639,293–639,403	fabG/WD0651	111
719,048–719,171	WD0744/WD0745	99
723,861–724,026	WD0749/WD0750	166
764,459–764,871	WD0790/WD0791	413
768,936–768,988	rho/WD0796	53
850,067–850,142	WD0878/trx	76
932,596–932,693	WD0973/WD0974	98
940,039–940,142	nuoI/trmE	104
941,823–941,975	tRNA-Ser-2/WD0982	153
1,039,579–1,039,870	WD1081/WD1082	292
1,105,661–1,105,744	tRNA-Thr-2/mutM	84

We then checked the read pairs mapping to these regions in the *w*MelPop-CLA RNA-Seq data, as described above. Reads were mapped to all but one of these intergenic regions, indicating that they were transcribed in this strain. However, only one of these intergenic regions showed evidence of specific transcription, rather than transcription as part of a unit with one or both flanking genes. This region (*w*Mel coordinates 1,039,579–1,039,870) was therefore selected for experimental validation together with the other candidate sRNAs identified using RNA-Seq data above.

This approach is limited to the comparison of strains with an intermediate level of divergence. We repeated the *changept* analysis comparing the genomes of two more closely related *Wolbachia* strains, *w*Mel and *w*Ri. These genomes have undergone only limited divergence, and we found that over 70% of the genome was assigned to the most highly conserved class of segments, greatly reducing the predictive power of the method. All highly conserved non-CDS regions identified in the *w*Mel-*w*Pip comparison were, however, also identified in the *w*Mel-*w*Ri comparison. At the other end of the taxonomic scale, we also attempted to repeat this analysis comparing the genomes of *w*Mel or *w*Pip with the more distantly related D group strain *w*Bm. Unfortunately, the genomes of these strains have undergone extensive rearrangement since their divergence, and too few genomic regions with conserved synteny and of sufficient length could be identified to perform the analysis.

### Experimental validation of candidate sRNAs

For all subsequent experiments to investigate expression of our candidate sRNAs, we used *Wolbachia*-infected insects, rather than cell culture, to ensure that our results reflect the natural biology of the symbiont. As an initial step, we tested whether the candidate sRNAs identified by our two methods were transcribed specifically, rather than as part of a single transcript with a flanking gene, in *w*Mel in *D*. *melanogaster*. We first used a 5’ RACE procedure to identify the 5’ end of those candidate sRNA transcripts for which it was possible to design a combination of RACE primers specific to the intergenic region. In many cases this was not possible, due to the high levels of repetitive sequence in the *w*Mel genome. These transcripts were discarded as candidates for further analysis.

We performed the 5'RACE procedure on 13 candidate sRNAs. Of these, five amplified successfully, and sequencing of the resulting plasmid showed that the 5' end of each RNA was indeed within the intergenic region (Table 4). The sequences of the plasmids are provided in Table C in S1 File. The other regions did not amplify, which could be due to multiple factors: no expression in the given biological conditions (age or tissue for example), expression below our detection limit, or expression from the opposite DNA strand as we designed all the primers on the positive strand only for this preliminary analysis. These non-amplifying regions were not considered further as candidate sRNAs.

**Table 4:** Intergenic regions (IGR) selected for 5’RACE experiments^a^ and name and position of the two putative*Wolbachia* small non-coding RNAs we identified.

Coordinates in *w*Mel genome	IGR size (bp)	Upstream/downstream CDS (IGR ID^b^)	5’ end coordinate^c^	3’ end within IGR?^d^	Name of putative sRNA
67,695–68,395	700	WD0072/WD0073 (IG-60)	67,860	Yes	*ncrwmel01*
170,838–171,549	711	WD0187/mutS (IG-151)	NA	NA	
461,742–462,763	1021	WD0478/WD0480 (IG-292)	NA	NA	
527,661–528,615	954	hemC/sucB (IG-446)	NA	NA	
587,733–588,439	706	WD0609/WD0610 (IG-498)	587,739	No	
896,038–896,357	319	WD0931/WD0932 (IG-760)	NA	NA	
915,181–915,500	319	WD0955/WD0956 (IG-781)	NA	NA	
978,741–979,093	352	WD1015/WD1016 (IG-834)	NA	NA	
1,039,579–1,039,870	291	WD1081/WD1082 (IG-884)	1,039,620	Yes	*ncrwmel02* ^e^
1,080,340–1,081,546	1206	tRNA-Arg-4/WD1131 (IG-921)	NA	NA	
1,189,867–1,190,417	550	WD1243/WD1244 (IG-1021)	1,190,012	No	
1,216,366–1,216,864	498	ispH/WD1275 (IG-1047)	1,216,539	NA	
1,217,297–1,217,892	595	WD1276/htpG (IG-1049)	NA	NA	

^a^Note that we did not demonstrate sRNA-like intergenic-specific transcription for most of these regions

^b^IGR ID as designed in the S1 Data

^c^Determined by 5’RACE

^d^Determined by RT-PCR with downstream CDS

^e^Also predicted by the bioinformatic approach.

In addition to performing 5'RACE, 'overlapping RT-PCR' was done to verify that the 3' end of each of the candidate sRNAs was also within the intergenic region. Of the five regions with a 5' end confirmed by 5'RACE to be within an intergenic region, it was possible to design specific RT primers for four. Two regions could be amplified using a forward intergenic primer and reverse downstream gene primer, indicating that the transcription of these regions occurred as a single RNA molecule with a flanking gene. These two intergenic regions might contain sRNAs transcribed as part of an operon, but could alternatively be 5' UTR regions, and so were excluded from further analysis. However, two regions showed no amplification when RT-PCR was performed using reverse primers in the downstream flanking gene, while amplification occurred using forward and reverse primers that both bound to the intergenic region. These results demonstrate that the transcription of RNA from these regions begins and ends in the intergenic region, and these two RNA molecules can consequently be considered to be intergenic sRNAs. We labeled them non-coding RNA *W* *olbachia* wMel 01 and 02 (*ncrwmel01* and *ncrwmel02*). The putative sRNA *ncrwmel02* is a highly conserved intergenic region that was predicted by both RNA-Seq and comparative genomics approaches (Table 4).

### Putative sRNA shows sequence conservation but differential transcript levels across *Wolbachia* strains

We then used qPCR to test for differences in the expression of *ncrwmel02* in four different *Wolbachia* strains (*w*Mel, *w*MelPop, *w*MelCS and *w*Au) in the whole body of 1 day-old male *D*. *melanogaster* flies. We selected this putative sRNA because its sequence is conserved (Figure C in S1 File), and we were able to design specific qPCR primers for the intended template and successfully amplify cDNA from all strains, while we could not for *ncrwmel01*.

**Fig 2:**
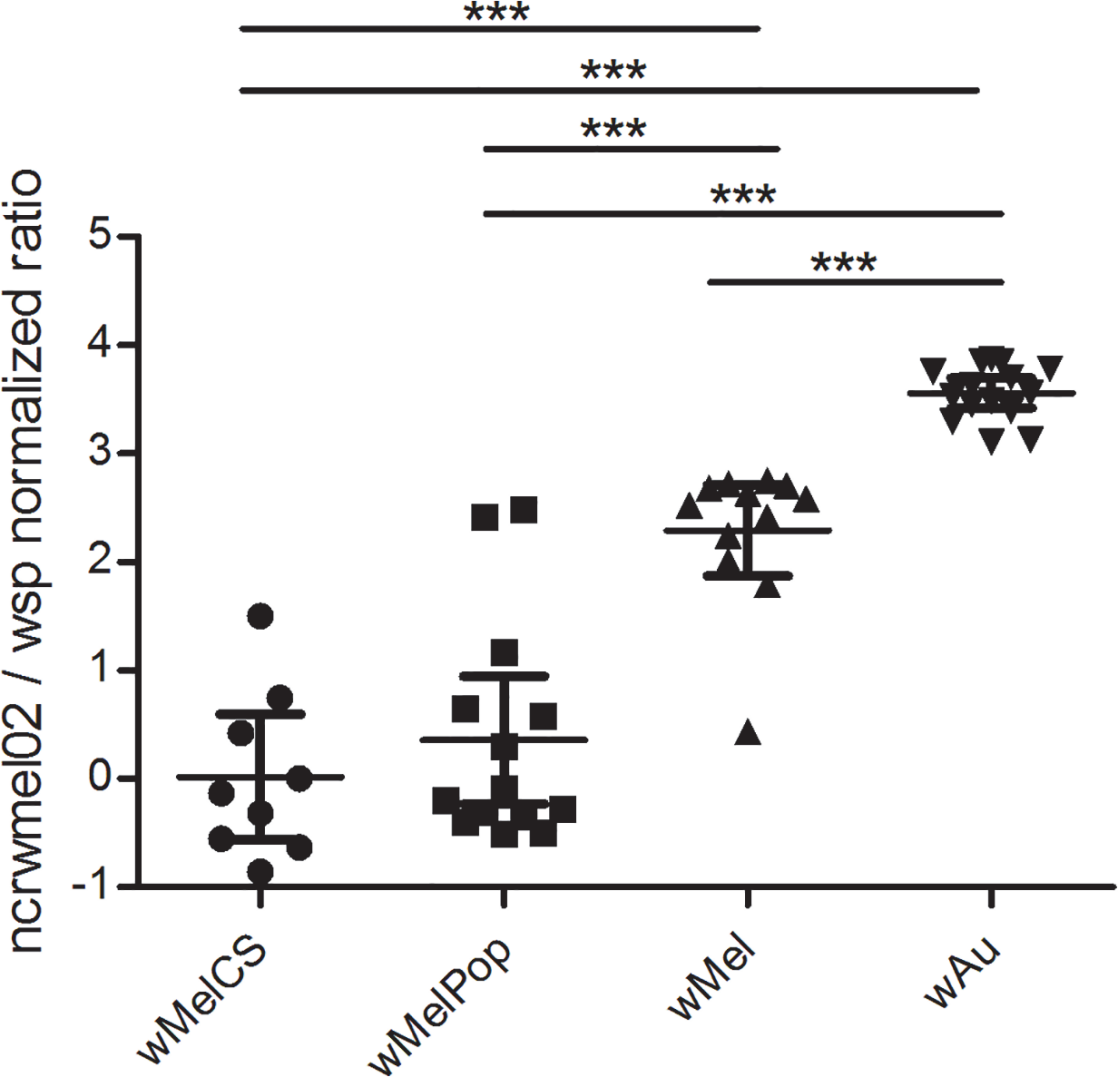
Expression of *ncrwmel02* in four *Wolbachia* strains from the whole body of 1-day-old male *D*. *melanogaster*. Expression (mean ± 95% CI) normalized to *wsp* expression (Mann-Whitney *U* test ** p < 0.01, **** p < 0.0001)

The expression of *ncrwmel02* was normalized against the expression of the *Wolbachia* Surface Protein encoding gene, *wsp*, to account for differences in *Wolbachia* density between the strains. *ncrwmel02* was expressed at a relatively low level in *w*MelCS and *w*MelPop, but approximately twice as highly in *w*Mel, and seven-fold more highly in *w*Au (Fig. 2).

### *Wolbachia* putative sRNA expression is differentially regulated in host tissues and sexes

**Fig 3:**
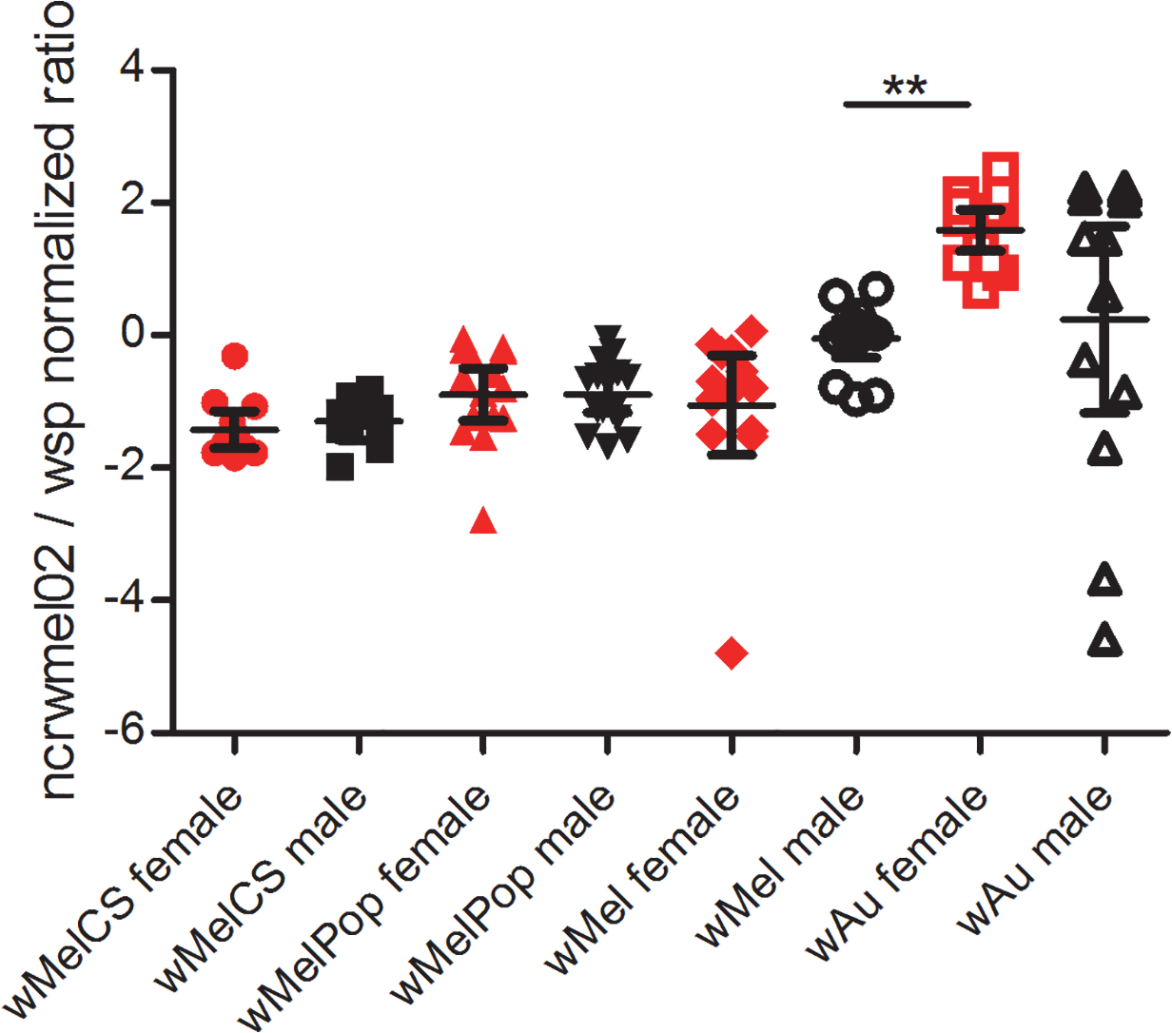
Expression of *ncrwmel02* in four *Wolbachia* strains from abdomens of 1-day old male (black) or female (red) *D*. *melanogaster*. Expression (mean ± 95% CI) normalized to *wsp* expression (Mann-Whitney *U* test, ** p < 0.01)

In order to assess whether the expression of this *Wolbachia* putative sRNA is constitutive or regulated, we analysed its expression in different tissues of male and female flies. First, *ncrwmel02* expression was compared in the abdomens of male and female 1-day-old flies for the four *Wolbachia* strains (Fig. 3). We observed only one significant difference in *ncrwmel02* expression in these tissues: *ncrwmel02* in *w*Mel was more highly expressed in male than in female abdomens, demonstrating that in some conditions its expression is differentially regulated.

**Fig 4:**
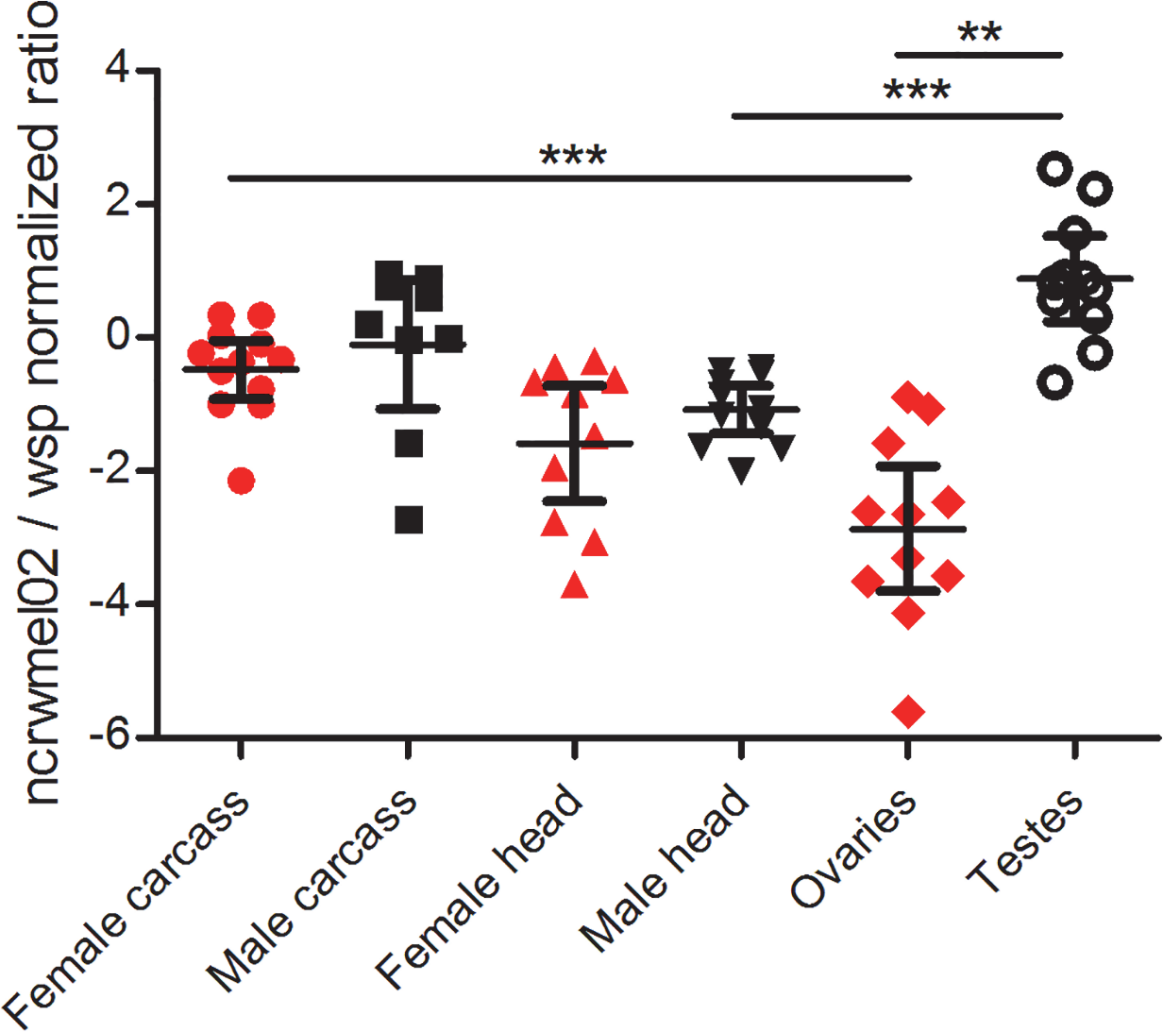
Expression of *ncrwmel02* in the *w*Mel strain from gonads, heads and carcasses of 1-day-old male (black) or female (red) *D*. *melanogaster*. Expression (mean ± 95% CI) normalized to *wsp* expression (Mann-Whitney *U* test, ** p < 0.01 *** p < 0.001)

Because *ncrwmel02* expression in whole abdomens might not reflect the level of regulation occurring in specific *Wolbachia*-infected tissues, we performed a second experiment in which its expression was analyzed in dissected tissues (gonads, head and carcasses) of male and female flies infected with the *w*Mel strain (Fig. 4). In contrast to our observation of generally stable *ncrwmel02* expression in male and female whole abdomens, its expression in dissected tissues showed clear evidence of differential expression.

The greatest differences were observed between gonads (ovaries and testes), which have very different physiological activity and regulation. Expression was significantly upregulated in testes compared to ovaries with more than ten-fold difference. For body parts that are expected to be more similar in terms of regulation and activity between males and females, such as heads and carcasses, no differential regulation of *ncrwmel02* in different host sexes was observed (Fig. 4). In addition, we also observed differential expression of *ncrwmel02* when comparing different tissues in the same sex. For example, expression was upregulated in female carcasses compared to female gonads, and upregulated in male gonads compared to male heads. To validate the differential expression we observed on those dissected tissues we compared the use of *wsp*, *Wolbachia* 16S and *Drosophila melanogaster rps17* as reference genes. Whatever the gene used as reference, all the patterns of expression remain the same and all differential expression remains significant (Figure D in S1 File).

In total, these expression experiments show that the *Wolbachia* putative sRNA *ncrwme02* is expressed in the four *Wolbachia* strains in a regulated pattern that differs according to the sex of the host and the tissue in which the bacterium is localized.

## Discussion

Recent research has begun to uncover the critical roles played by small RNAs in the regulation of cellular processes ranging from highly conserved housekeeping functions to rapid responses to environmental or host cues. Most research on sRNAs to date has focused on free-living or facultatively intracellular bacteria. Yet we might expect that obligately host-associated bacteria would rely on sRNAs at least as much as, if not more than, free-living bacteria, for two reasons. First, sRNAs offer bacteria a flexible and rapidly adaptable mode of gene regulation that may be ideally suited to the constantly changing co-adaptive interplay between host and symbiont. Secondly, many endosymbiotic bacteria, including *Wolbachia*, have undergone at least some degree of genome reduction, often resulting in the loss of genes encoding canonical transcriptional regulatory proteins [18]. Intergenic regulatory regions associated with sRNAs show evidence of retention and conservation even in some of the most reduced endosymbiotic genomes [57], indicating that sRNA-based regulation may remain necessary and be under sufficient selection to resist loss via genome reduction.

We have identified two novel putative sRNAs in *Wolbachia* genomes, using two independent methods that are likely to detect different subsets of sRNAs. The comparative genomics approach we used could detect sRNA candidates that are conserved at the nucleotide level across strains from A and B supergroups of *Wolbachia*, regardless of the conditions under which they are expressed. It would not, however, identify candidates that are not present in both strains, have originated since the divergence of the supergroups, or are conserved at the level of secondary structure rather than nucleotide sequence. In contrast, RNA-Seq data could be used to detect these latter classes of sRNAs, but would not be able to identify sRNAs if they were not expressed under the experimental conditions used to generate the data. The two putative sRNAs characterized here were identified by our analysis of *w*MelPop-CLA RNA-Seq data, while only *ncrwmel02* was predicted using comparative genomics. This probably reflects the level of sequence conservation of these putative sRNAs: when used as a blastN query against the NCBI NT and WGS databases, *ncrwmel02* has longer hits, with higher percentage sequence identity, to a broader range of other *Wolbachia* strains (from the A, B, C and D supergroups), than *ncrwmel01*.

We showed *ncrwmel02* was present and transcribed in the four A group strains we used to experimentally characterize the expression of this putative sRNA. The strains *w*Mel, *w*MelCS and *w*MelPop are closely related [58], naturally infect *D*. *melanogaster*, and all induce host cytoplasmic incompatibility (CI), the most frequently observed type of reproductive manipulation caused by *Wolbachia*. *w*Mel and *w*MelCS are otherwise benign, but *w*MelPop is pathogenic, causing its adult hosts to die prematurely. In contrast, *w*Au is somewhat more distantly related [59], infects *D*. *simulans*, and does not cause CI. All four strains were placed into the same genetic background (*D*. *melanogaster w* ^*1118*^) for these analyses, to limit the effects of different host species on sRNA expression patterns.

Almost all pairwise strain comparisons are significantly different at the whole body level for *ncrwmel02* expression; most strikingly, it is substantially more highly expressed in *w*Au than in the three other strains. Host sex-specific differences in expression in *w*Mel become apparent at the tissue level. *ncrwmel02* is more highly expressed in testes than in ovaries, but does not show evidence of male-specific upregulation in other tissues. The significant upregulation of *ncrwmel02* in testes compared to ovaries suggests that sRNAs might possibly be involved in some aspect of host reproductive manipulation, although the data we provide here only suggest this hypothesis and future experimental demonstration would be required on a range of CI and non-CI inducing strains. CI involves *Wolbachia*-induced modification of sperm in infected hosts [2], and increased transcription of sRNAs may play a role in that process. More generally, given the regulatory roles of sRNAs in other bacterial species, differential expression of sRNA could contribute to a range of phenotypic differences between strains.

Developing a full understanding of the roles of sRNA in *Wolbachia* will require not only searching for additional sRNAs and characterizing their expression in different strains and host tissues, but identifying the targets of these molecules. Many of these targets, whether genes, mRNA or proteins, are expected to be of *Wolbachia* origin, but it is also possible that *Wolbachia* sRNAs could directly target host gene expression. Although secretion of functional bacterial sRNAs into eukaryotic host cells has not been observed, it is a possibility worth considering [60]. Viral sRNAs are known to target host genes [61], and bacterial sRNAs may have the same ability. In addition, it has already been shown that interplay occurs between *Wolbachia* and its insect host via eukaryote miRNA [8,9], and that the virus-blocking phenotype induced by *Wolbachia* involves changes in the expression of host miRNA [10]. It is possible that *Wolbachia*-induced phenotypes such as dengue inhibition may occur as part of a molecular dialogue between the bacterial endosymbiont and its eukaryotic host involving uni- or bi-directional gene regulation by small non-coding RNAs.

## Conclusions

Considering (a) the fundamental roles played by sRNA in other bacteria, especially in quorum-sensing, pathogenesis and virulence, (b) the conservation of *ncrwmel02* between different *Wolbachia* strains, and (c) the strain-, sex- and tissue-specific differential regulation of *ncrwmel02* expression, we hypothesize that sRNAs may play significant roles in the biology of *Wolbachia*. The analyses described here are preliminary and had limited power, and the two putative sRNAs we have identified are likely to represent only the largest, most highly expressed and/or conserved sRNAs in *Wolbachia* genomes. Additional RNA-Seq experiments with different size selection of RNA, bacterial purification, host and rRNA depletion [49,50], and strand-specific library preparation might allow the identification of many more of these molecules, and further research will be required to assess the roles of sRNAs in the insect-*Wolbachia* interaction. Nonetheless, the descriptive work presented here opens a new path in understanding the molecular mechanisms underlying the complex and diverse range of phenotypes induced by *Wolbachia* within its host.

## Supporting Information

S1 Data RNA-Seq reads mapping to *w*Mel features. Reads mapping to CDS and intergenic regions are indicated for all 5 RNA-Seq experiments.
(XLS)

S1 File Figure A, Selection of optimal number of classes. We used approximations to the well-known information criteria AIC, BIC and DIC to identify the number of distinct classes of conservation levels. Generally, a lower value of the information criteria indicates a better model. BIC favoured a 1-class model, which is inappropriate. We therefore based our judgement on AIC and DICV and selected the 7-class model as the first local minimum of AIC and DICV has occurred at seven classes. **Figure B, Identifying the most conserved class**. The mean proportion of alignment matches was plotted against each iteration of the sampler to identify the class that contains the most conserved segments in *w*Mel and *w*Pip (Class 4). The different colours represent different classes in the 7-class model. **Figure C, Sequence alignment of the *ncrwmel02* amplicon from the published genome data of *w*Mel [18], *w*MelCS, *w*MelPop [58] and *w*Au [62]. Figure D, Validation of *ncrwmel02* differential expression observed using *wsp* as reference gene in dissected tissues of *w*Mel-infected male (black) or female (red) *D*. *melanogaster***. *ncrwmel02* expression calculated using *wsp*, 16S or *rps17* is represented for the three significant differential expression observed using *wsp*. Expression (mean ± 95% CI) normalized to *wsp*, 16S or *rps17* expression (Mann-Whitney U test, * p < 0.1, ** p < 0.01 *** p < 0.001). Panel A: *ncrwmel02* expression in male and female gonads. Panel B: *ncrwmel02* expression in female dissected tissues. Panel C: *ncrwmel02* expression in male dissected tissues. **Table A, Oligonucleotides used in this study. Table B, Highly conserved non-coding region predicted by changept**. Thresholds used: 1. Conservation = 0.95 (conservation level of the most conserved class-Class 4); 2. Profile value ≥0.5 (probability that each position in the conserved feature belongs to Class 4); 3. Length >50 nt (length of the conserved feature). **Table C, 5’ RACE of intergenic regions (IGR) plasmid sequences**. Insert in pGEMTeasy in bold.
(DOC)
