# Supplementary material for: Discovery of Putative Small Non-Coding RNAs from the Obligate Intracellular Bacterium Wolbachia pipientis
Source: PLoS One. 2015 Mar 4;10(3):e0118595. doi: 10.1371/journal.pone.0118595 (PMC4349823; doi:10.1371/journal.pone.0118595)
Supplement: S1 File — We used approximations to the well-known information criteria AIC, BIC and DIC to identify the number of distinct classes of conservation levels. Generally, a lower value of the information criteria indicates a better model. BIC favoured a 1-class model, which is inappropriate. We therefore based our judgement on AIC and DICV and selected the 7-class model as the first local minimum of AIC and DICV has occurred at seven classes. Figure B, Identifying the most conserved class. The mean proportion of alignment matches was plotted against each iteration of the sampler to identify the class that contains the most conserved segments in wMel and wPip (Class 4). The different colours represent different classes in the 7-class model. Figure C, Sequence alignment of the ncrwmel02 amplicon from the published genome data of wMel [18], wMelCS, wMelPop [58] and wAu [62]. Figure D, Validation of ncrwmel02 differential expression observed using wsp as reference gene in dissected tissues of wMel-infected male (black) or female (red) D. melanogaster. ncrwmel02 expression calculated using wsp, 16S or rps17 is represented for the three significant differential expression observed using wsp. Expression (mean ± 95% CI) normalized to wsp, 16S or rps17 expression (Mann-Whitney U test, * p < 0.1, ** p < 0.01 *** p < 0.001). Panel A: ncrwmel02 expression in male and female gonads. Panel B: ncrwmel02 expression in female dissected tissues. Panel C: ncrwmel02 expression in male dissected tissues. Table A, Oligonucleotides used in this study. Table B, Highly conserved non-coding region predicted by changept. Thresholds used: 1. Conservation = 0.95 (conservation level of the most conserved class-Class 4); 2. Profile value ≥0.5 (probability that each position in the conserved feature belongs to Class 4); 3. Length >50 nt (length of the conserved feature). Table C, 5’ RACE of intergenic regions (IGR) plasmid sequences. Insert in pGEMTeasy in bold. (DOC) [file pone.0118595.s002.doc]

**Fig A. Selection of optimal number of classes.** We used approximations to the well-known information criteria AIC, BIC and DIC to identify the number of distinct classes of conservation levels. Generally, a lower value of the information criteria indicates a better model. BIC favoured a 1-class model, which is inappropriate. We therefore based our judgement on AIC and DICV and selected the 7-class model as the first local minimum of AIC and DICV has occurred at seven classes.

**Fig B. Identifying the most conserved class.** The mean proportion of alignment matches was plotted against each iteration of the sampler to identify the class that contains the most conserved segments in *w*Mel and *w*Pip (Class 4). The different colours represent different classes in the 7-class model.

wMel TGTAGCGTTATGAATTAGGAGTGCTATATTAAAGCTTACCTCACTATTAAAGCTATCGGTCAGATTAGATTAAAAACCTAATCTGACCGGTTTC

wMelCS tgtagcgttatgaattaggagtgctatattaaagcttacctcactattaaagctatcggtcagattagattaaaaacctaatctgaccggtttc

wMelPop tgtagcgttatgaattaggagtgctatattaaagcttacctcactattaaagctatcggtcagattagattaaaaacctaatctgaccggtttc

wAu TGTAGCGTTATGAATTAGGAGTGCTATATTAAAGCTTACCTCACTATTAAAGCTGTCGGTCAGATTAGATTAAAAACCTAATCTGACCGGTTTC

**Fig. C. Sequence alignment of the *ncrwmel02* amplicon from the published genome data of *w*Mel (Wu et al 2004), *w*MelCS, *w*MelPop (Woolfit et al 2013) and *w*Au (Sutton et al 2014).**


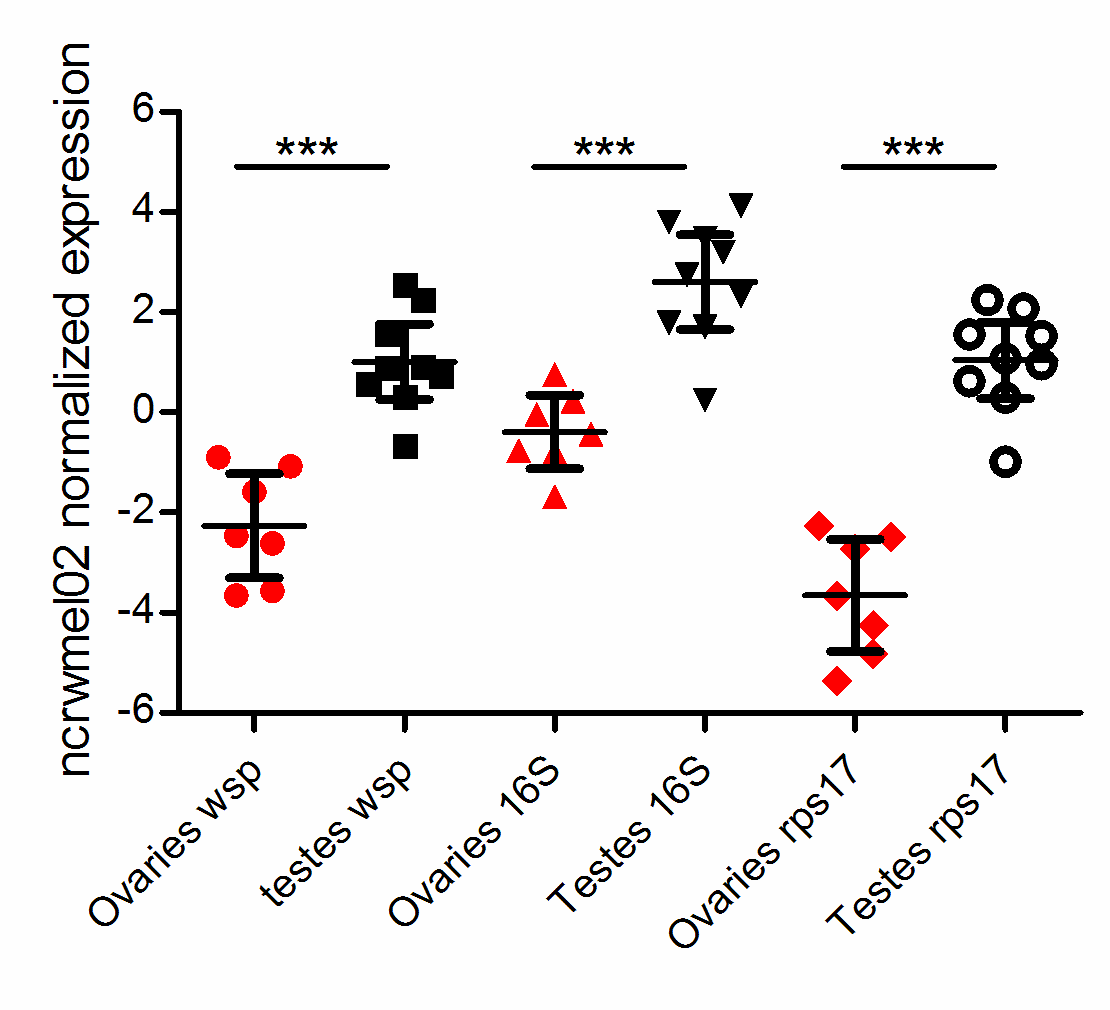

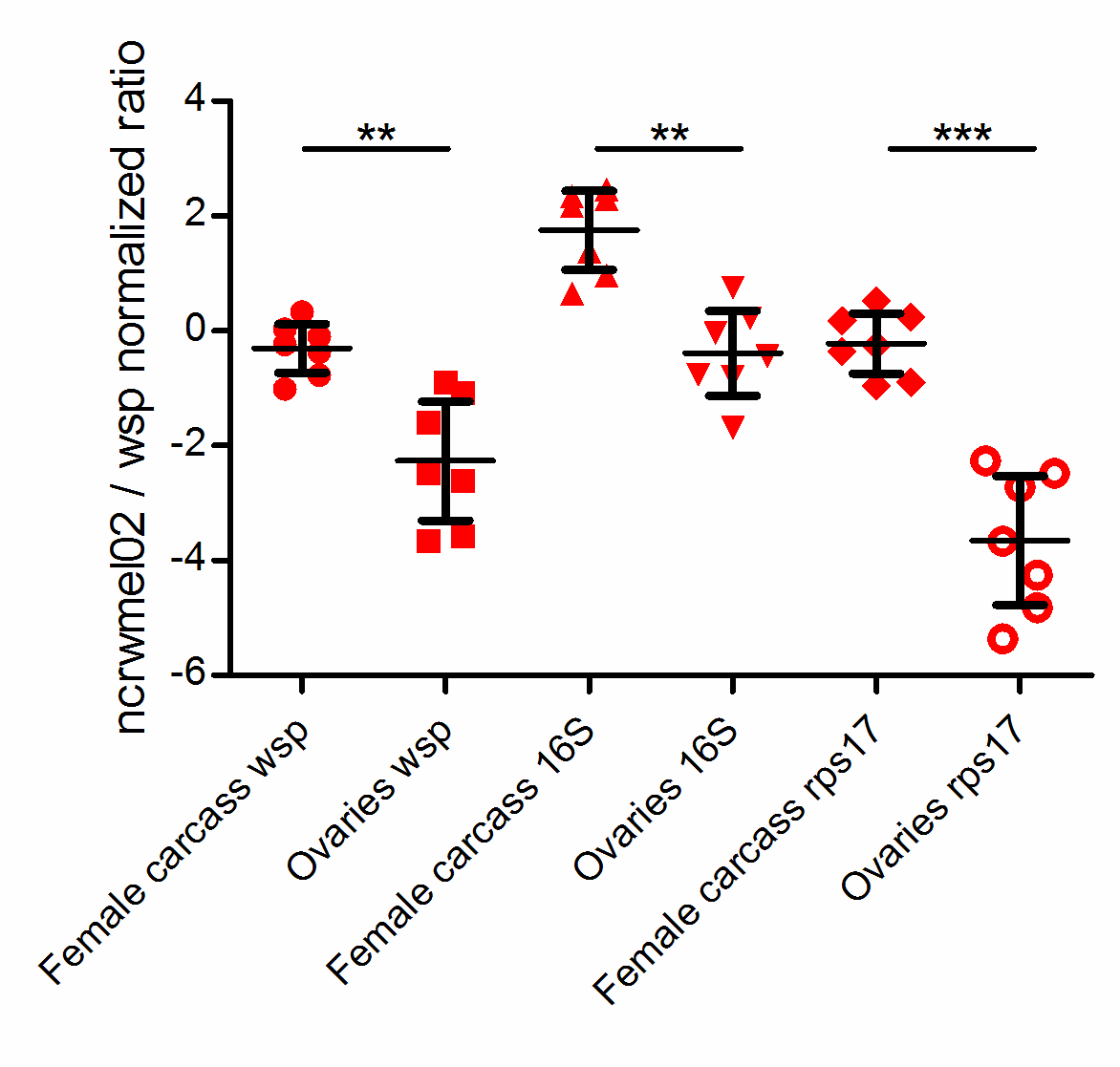

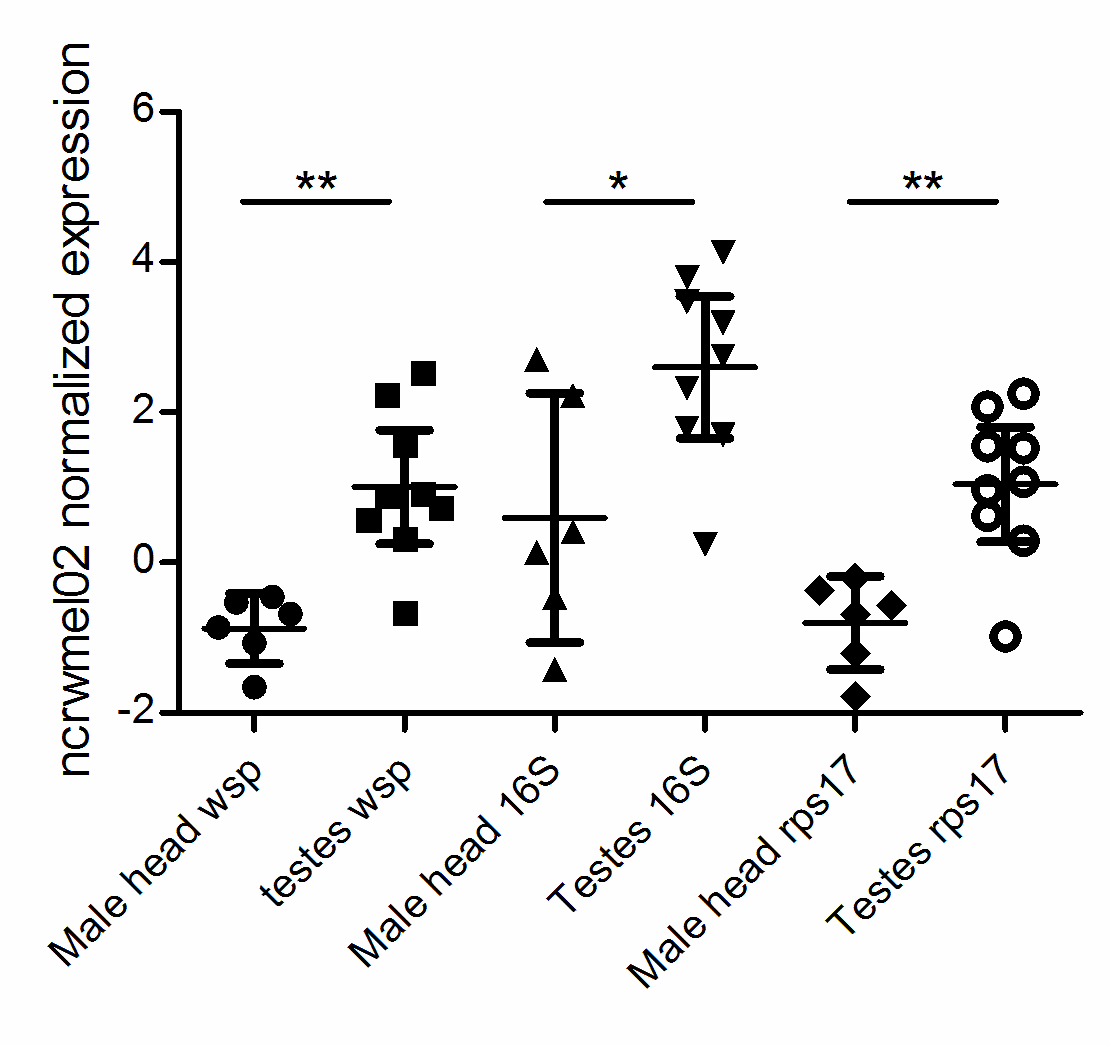
**A B C**

**Fig. D. Validation of *ncrwmel02* differential expression observed using *wsp* as reference gene in dissected tissues of *w*Mel-infected male (black) or female (red) *D. melanogaster*.** *ncrwmel02* expression (mean ± 95% CI) normalized to *wsp*, 16S or *rps17* expression (Mann-Whitney U test, * p < 0.1, ** p < 0.01 *** p < 0.001). Panel A: *ncrwmel02* expression in male and female gonads. Panel B: *ncrwmel02* expression in female dissected tissues. Panel C: *ncrwmel02* expression in male dissected tissues

**Table A. Oligonucleotides used in this study**.

| Sequence (5’-3’) | Description | Reference |
| --- | --- | --- |
| **5’RACE primers** |  |  |
| AUAUGCGCGAAUUCCUGUAGAACGAACACUAGAAGAAA | RNA adaptor |  |
| GCGCGAATTCCTGTAGA | Adaptor specific PCR primer |  |
|  |  |  |
| GGATCTATGTTAAGAGATACCGTGAA | IGR-60-specific RT primer | This study |
| ATGACGGTTCGTGACGGTAT | IGR-60-specific PCR primer | This study |
|  |  |  |
| gcagcttaatcttgcttgtca | IGR-151-specific RT primer | This study |
| acgccaatattttaaagcggata | IGR-151-specific PCR primer | This study |
|  |  |  |
| gcaaaagaagccctgaggtt | IGR-392-specific RT primer | This study |
| AGAAGCCCTGAGGTTATTATCCGCT | IGR-392-specific PCR primer | This study |
|  |  |  |
| TCGCACTACGTGCATCGCAT | IGR-446-specific RT primer | This study |
| ctacgtgcatcgcatgtctt | IGR-446-specific PCR primer | This study |
|  |  |  |
| TTTCAAGCTTTGCCAAAAGAA | IGR-498-specific RT primer | This study |
| CCCCAATCAAAACAGCCTTA | IGR-498-specific PCR primer | This study |
|  |  |  |
| CACTTGAGCGATGCAACAAAGCCA | IGR-760-specific RT primer | This study |
| aacaaagccatcccagtgtc | IGR-760-specific PCR primer | This study |
|  |  |  |
| atgggagggaagcaaaatct | IGR-781-specific RT primer | This study |
| GGGAAGCAAAATCTGGCTTAATGGC | IGR-781-specific PCR primer | This study |
|  |  |  |
| ttgcatgacaccctgacaac | IGR-834-specific RT primer | This study |
| gctacgtgttagcgggatct | IGR-834-specific PCR primer | This study |
|  |  |  |
| CGCTCGTGCACAAATTAAAA | IGR-884-specific RT primer | This study |
| TGTAGCGTTATGAATTAGGAGTGC | IGR-884-specific PCR primer | This study |
|  |  |  |
| CATAGATCCCGCTAACACGTAG | IGR-921-specific RT primer | This study |
| agccccgtggttattatctg | IGR-921-specific PCR primer | This study |
|  |  |  |
| CCCGTGTTAGCTAGTTGTCACTCCC | IGR-1021-specific RT primer | This study |
| ATCCTGCAAATTGGCGTACT | IGR-1021-specific PCR primer | This study |
|  |  |  |
| agcagtgggatgacgagact | IGR-1035-specific RT primer | This study |
| AAAGAAGCCCCGTGGTTGGC | IGR-1035-specific PCR primer | This study |
|  |  |  |
| CGAGATTCAGCCGCTTTTA | IGR-1047-specific RT primer | This study |
| GCAACTAACCTACGCTGCAA | IGR-1047-specific PCR primer | This study |
|  |  |  |
| tgtatttggcgtaaatcatgc | IGR-1049-specific RT primer | This study |
| gcactatgtgcacctcatgtct | IGR-1049-specific PCR primer | This study |
|  |  |  |
| **RT-PCR primers** |  |  |
| TGGATCCCAGTGTCAAGCAC | IGR-60 Fwd in IGR | This study |
| ACGACAATCGTCATCCCAGC | IGR-60 Rev in downstream CDS | This study |
| CGACGGCATGACGATAAGGT | IGR-60 Rev in IGR | This study |
|  |  |  |
|  |  |  |
| GCTGTTTTGATTGGGGTCTT | IGR-498 Fwd in IGR | This study |
| TCGTATCGGGCAAGAACGTA | IGR-498 Rev in downstream CDS | This study |
| TTTCAAGCTTTGCCAAAAGAA | IGR-498 Rev in IGR | This study |
|  |  |  |
| GAAACCGGTCAGATTAGGTTTTT | IGR-884 Fwd in IGR | This study |
| CCGTAACCGGCACTGAAGTA | IGR-884 Rev in downstream CDS | This study |
| TGTAGCGTTATGAATTAGGAGTGC | IGR-884 Rev in IGR | This study |
|  |  |  |
| AACAACGTAGTTGGCGTCTT | IGR-1021 Fwd in IGR | This study |
| AGCACTGGGATGACACCATT | IGR-1021 Rev in downstream CDS | This study |
| AACAACGTAGTTGGCGTCTT | IGR-1021 Rev in IGR | This study |
|  |  |  |
| **qPCR primers** |  |  |
| Fwd GAAACCGGTCAGATTAGGTTTTT | *ncrwmel02*qPCR primers | This study |
| Rev TGTAGCGTTATGAATTAGGAGTGC |  |  |
|  |  |  |
| Fwd ATCTTTTATAGCTGGTGGTGGT | *wsp* qPCR primers |  |
| Rev GGAGTGATAGGCATATCTTCAAT |  |  |
|  |  |  |
| Fwd CGGTGAATACGTTCTCGGGTC | *16S* qPCR primers | This study |
| Rev CACCCCAGTCACCGATCCC |  |  |
|  |  |  |
| Fwd CACTCCCAGGTGCGTGGTAT | *rps17* qPCR primers |  |
| Rev GGAGACGGCCGGGACGTAGT |  |  |

**Table B. Highly conserved non CDS predicted by *changept**.**

| **wMel coordinates** | **Type of conserved feature** | **Profile value** | **Length (nt)** |
| --- | --- | --- | --- |
| 1,739-2,162 | pseudo WD0002 | 1.0 | 424 |
| 2,274-2,503 | pseudo WD0002 | 0.5 | 230 |
| 3,024-3,118 | tRNA | 0.5 | 95 |
| 44,380-44,468 | intergenic | 1.0 | 89 |
| 83,877-83,957 | tRNA | 0.7 | 81 |
| 85,867-85,929 | intergenic | 1.0 | 63 |
| 117,042-117,328 | ncRNA tmRNA | 0.6 | 287 |
| 124,753-124,836 | tRNA | 0.9 | 84 |
| 182,216-185,396 | rRNA 23S+5S | 1.0 | 3181 |
| 279,526-279,619 | intergenic | 0.9 | 94 |
| 372,011-372,117 | tRNA | 0.8 | 107 |
| 513,727-513,814 | tRNA | 0.9 | 88 |
| 547,479-547,732 | intergenic | 1.0 | 254 |
| 611,202-611,370 | intergenic | 1.0 | 169 |
| 612,281-612,391 | intergenic | 0.7 | 111 |
| 622,779-622,923 | intergenic | 0.5 | 145 |
| 623,094-623,293 | intergenic | 1.0 | 200 |
| 639,293-639,403 | intergenic | 1.0 | 111 |
| 706,682-707,007 | ncRNA rnpB1 | 0.9 | 326 |
| 719,048-719,171 | intergenic | 0.5 | 99 |
| 722,484-722,594 | tRNA | 0.8 | 111 |
| 723,861-724,026 | intergenic | 1.0 | 166 |
| 764,459-764,871 | intergenic | 1.0 | 413 |
| 768,936-768,988 | intergenic | 0.7 | 53 |
| 793,553-793,636 | tRNA | 0.8 | 84 |
| 840,429-840,513 | tRNA | 1.0 | 85 |
| 850,067-850,142 | intergenic | 0.9 | 76 |
| 932,596-932,693 | intergenic | 0.5 | 98 |
| 934,908-935,042 | tRNA | 1.0 | 135 |
| 935,321-935,403 | tRNA | 0.8 | 83 |
| 940,039-940,142 | intergenic | 1.0 | 104 |
| 941,714-941,808 | tRNA | 0.6 | 95 |
| 941,823-941,975 | intergenic | 1.0 | 153 |
| 970,671-970,776 | tRNA | 1.0 | 106 |
| 1,039,579-1,039,870 | intergenic | 1.0 | 292 |
| 1,105,661-1,105,744 | intergenic | 0.9 | 84 |
| 1,107,303-1,107,403 | tRNA | 1.0 | 101 |
| 1,152,142-1,152,229 | tRNA | 0.7 | 88 |
| 1,158,600-1,158,694 | tRNA | 1.0 | 95 |
| 1,167,332-1,169,526 | rRNA 16S | 1.0 | 2195 |
| 1,186,283-1,186,373 | tRNA | 1.0 | 91 |
| 1,208,797-1,208,903 | tRNA | 0.5 | 107 |

* Thresholds used: 1. Conservation = 0.95 (conservation level of the most conserved class-Class 4); 2. Profile value ≥0.5 (probability that each position in the conserved feature belongs to Class 4); 3. Length >50 nt (length of the conserved feature)

**Table C. 5’ RACE of intergenic regions (IGR) plasmid sequences*.**

| IGR coordinates in wMel genome | Sequence |
| --- | --- |
| IGR-60 | CACTAGTGATT**GCGCGAATTCCTGTAGAACGAACATTAGAAGAAAAAAAAACATTGTATTTTAACGTAAAACAGCTATTTTTATGCTCACCAACTTAATAAAATTCCTGGATCCCAGTGTCAAGCACTGGGATGACAAGATATAAACCTTATCGTCATACCGTCACGAACCGTCAT**AATCGAATTCCCGCGGCCGCC |
| IGR-498 | CACTAGTGATT**GCGCGAATTCCTGTAGACTAGAAGAAAAAAAACTTCTTTTAAGATTAAGCGTTTGAAAGGTTTTTGAGTAAGGCTGTTTTGATTGGGG**AATCGAATTCCCGCGGCCGCC |
| IGR-884 | CACTAGTGATT**GCGCGAATTCCTGTAGAACACGAAGAAAAGAGTTTAGAGGGTTATAGAGAAACCGGTCAGATTAGGTTTTTAATCTAATCTGACCGATAGCTTTAATAGTGAGGTAAGCTTTAATATAGCACTCCTAATTCATAACGCTACA**AATCGAATTCCCGCGGCCGCC |
| IGR-1021 | CACTAGTGATT**ATCCTGCAAATTGGCGTACTATACTGTCTTAAACGACTTATAAGCGCGTTTCAGCTTGTGCAGGTAAAAACCTAGAATATTGTGAAGACATAAGGTGCACATAGTGCAAAAAATTAAAAATAAGACGCCAACTACGTTGTTTTCTTGCTGTTTAATCTGCACAGATGAAGATAACTGAATGCCTTCTTTCTTCTAGTTTCTACAGG**AATTCGCGCAATCGAATTC  CCGCGGCCGCC |
| IGR-1047 | GGCCGCGGGAATTCGATT**GCGCGAATTCCTGTAGAATAGAAGAAAGAAGCTATTGTATTTGCTTTCGCCAATCTGCAGATTAAAAGGTAAGGATTACTTAATGTATCGGCGTCTTATGTTCAATTTTTTGCAGTATATAGATACTGTATGTCTTTACAAAACTTCATCTACATCTAGATTTTTATCTAAATAAGCTGAACGCGCTTATAAAGCGTTACAAGACGTCAAAAAATGCCAATACTCGACAGAGATAGTAAAAGACTAGCTAACTCGGGGATTCTTTGTCTTTTTTTCTGCTTAGTAAATTTCTTAAACATTTGCAGCGTAGGTTAGTTGCA**ATCACTAGTGAATTCGCG |

* insert in pGEMTeasy in bold
